# HLA-G Expression in Human Mesenchymal Stem Cells (MSCs) Is Related to Unique Methylation Pattern in the Proximal Promoter as well as Gene Body DNA

**DOI:** 10.3390/ijms21145075

**Published:** 2020-07-18

**Authors:** B. Linju Yen, Hsiao-Lin Hwa, Pei-Ju Hsu, Pei-Min Chen, Li-Tzu Wang, Shih-Sheng Jiang, Ko-Jiunn Liu, Huey-Kang Sytwu, Men-Luh Yen

**Affiliations:** 1 Regenerative Medicine Research Group, Institute of Cellular & System Medicine, National Health Research Institutes (NHRI), Zhunan 350, Taiwan; hsulily@nhri.edu.tw; 2 Department of Obstetrics/Gynecology, Cathay General Hospital Shiji, Taipei 221, Taiwan; 3 Department of Obstetrics/Gynecology, National Taiwan University (NTU) Hospital & College of Medicine, Taipei 100, Taiwan; hwahl013@ntu.edu.tw (H.-L.H.); pmchen@ntu.edu.tw (P.-M.C.); lizwang62@gmail.com (L.-T.W.); 4 Institute of Forensic Medicine, College of Medicine, NTU, Taipei 100, Taiwan; 5 National Institute of Cancer Research, NHRI, Zhunan 350, Taiwan; ssjiang@nhri.edu.tw (S.-S.J.); kojiunn@nhri.edu.tw (K.-J.L.); 6 National Institute of Infectious Diseases & Vaccinology, NHRI, Zhunan 350, Taiwan; sytwu@nhri.edu.tw; 7 Graduate Institute of Microbiology and Immunology, National Defense Medical Center, Taipei 114, Taiwan

**Keywords:** mesenchymal stem cells (MSCs), human, HLA-G, embryonic stem cells (ESCs), bone marrow (BM), placenta, interferon-γ (IFN-γ), DNA methylation, promoter, gene body

## Abstract

Multipotent human mesenchymal stem cells (MSCs) harbor clinically relevant immunomodulation, and HLA-G, a non-classical MHC class I molecule with highly restricted tissue expression, is one important molecule involved in these processes. Understanding of the natural regulatory mechanisms involved in expression of this elusive molecule has been difficult, with near exclusive reliance on cancer cell lines. We therefore studied the transcriptional control of HLA-G in primary isolated human bone marrow- (BM), human embryonic stem cell-derived (hE-), as well as placenta-derived MSCs (P-MSCs), and found that all 3 types of MSCs express 3 of the 7 HLA-G isoforms at the gene level; however, fibroblasts did not express HLA-G. Protein validation using BM- and P-MSCs demonstrated expression of 2 isoforms including a larger HLA-G-like protein. Interferon-γ (IFN-γ) stimulation upregulated both gene and protein expression in MSCs but not the constitutively expressing JEG-3 cell line. Most interestingly in human MSCs and placental tissue, hypomethylation of CpG islands not only occurs on the HLA-G proximal promoter but also on the gene body as well, a pattern not seen in either of the 2 commonly used choriocarcinoma cell lines which may contribute to the unique HLA-G expression patterns and IFN-γ-responsiveness in MSCs. Our study implicates the importance of using normal cells and tissues for physiologic understanding of tissue-specific transcriptional regulation, and highlight the utility of human MSCs in unraveling the transcriptional regulation of HLA-G for better therapeutic application.

## 1. Introduction

Mesenchymal stem cells (MSCs) are somatic progenitors with multilineage differentiation capacity as well as strong immunomodulatory properties [1,2]. First isolated from the adult bone marrow (BM), MSCs have subsequently been found in numerous sources of tissues/organs [3]. The diverse biological properties of these easily accessible progenitors render MSCs as a popular source for cell therapy. MSC immunomodulation in particular has become increasingly important for clinical application since these properties have been shown to be broad, affecting multiple populations of leukocytes ranging from adaptive immune cells such as CD4 and CD8 T lymphocytes, to innate immune cells including dendritic cells, monocytes, macrophages, and myeloid-lineage cells [1,4,5]. However, these clinical trials have not yielded consistent results and hence have fueled continued interest in elucidating the mechanisms involved in MSC immunodulation to better tailor use of these progenitors to specific disease entities [6,7].

One of the most unique molecules by which human MSCs suppress effector leukocyte function is through the human leukocyte antigen G (HLA-G), a non-classical major histocompatibility complex (MHC) class I molecule [8]. First identified in placental trophoblasts, HLA-G is known to play an important role in mediating maternal tolerance of the fetus [9,10]. In contrast with classical MHC I molecules, HLA-G shows limited polymorphism and restricted tissue distribution, being expressed mainly by extravillous cytotrophoblasts with some expression detected in immune-privileged adult tissues such as the thymus and cornea [11,12,13]. HLA-G has unique alternative splicing patterns which are expressed as 7 different isoforms, including 4 membrane-bound (HLA-G1 to G 4) and 3 soluble forms (HLA-G5 to G7) [14,15]. Soluble HLA-G (sHLA-G) has been particularly relevant in the clinical setting, correlating with allograft transplantation tolerance and tumor escape [8]. HLA-G involvement in MSC immunodulation has been reported for adaptive/CD4 T cell responses [16,17,18,19]; moreover, developmentally early sources of MSCs have been found to express HLA-G protein that have functional relevance in suppressing innate leukocyte responses [20,21].

The restricted expression pattern of HLA-G has meant that its transcriptional regulation has been difficult to study, with data almost exclusively based on cancer cell lines and/or overexpression studies which collectively demonstrate many regulatory elements—including interferon-stimulated response element (ISRE) which can be activated by interferon-γ (IFN-γ), one of the most potent inducers of MHC class I proteins—on the proximal promoter of HLA-G are largely non-functional [22,23,24]. It is increasingly clear that epigenetic regulation is critical in defining tissue-specific gene expression patterns [25], with one of the most important epigenetic mechanisms controlling tissue-specific gene expression being methylation of genetic DNA which alters the local landscape without changing the DNA sequence to affect the binding ability of proteins [26]. Methylation occurs mainly at CG-rich regions which are blocks of ~1000 bp regions—also known as CpG islands—often found near gene promoters [27], and methylation particularly at CpG islands within the promoter region has been linked to transcriptional silencing while demethylation results in transcriptional activation [28]. Given the clinical relevance of HLA-G in immunomodulation overall and human MSC immunobiology, we therefore sought to understand the role of DNA methylation in transcriptional regulation of this molecule using primary isolated BM-MSCs, placenta-dervied MSCs (P-MSCs), human embryonic stem cell-derived MSCs (hE-MSCs), and term placental tissue.

## 2. Results

### 2.1. Diverse Sources of Human MSCs but Not Fibroblasts Express Multiple HLA-G mRNA and Protein Isoforms

**Figure 1:**
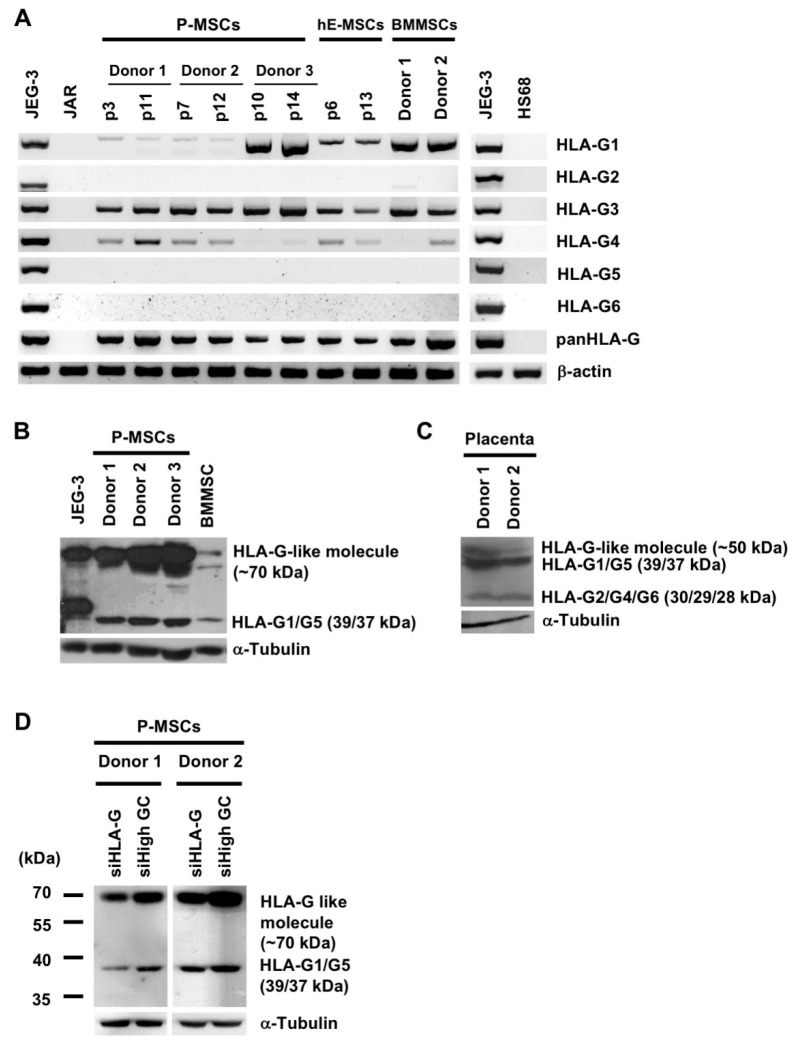
Diverse sources of human mesenchymal stem cells (MSCs) but not fibroblasts express multiple HLA-G mRNA and protein isoforms. (**A**) Expression of HLA-G mRNA variants in JEG-3 cells, JAR cells, human placenta-derived MSCs (P-MSCs), human embryonic stem cell-derived MSCs (hE-MSCs), bone marrow (BM) MSCs, and HS68 fibroblast cell line as analyzed by nested RT-PCR; “p” denotes passage number. (**B**) Expression of HLA-G protein isoforms in JEG-3 cells, P-MSCs, BMMSCs, and (**C**) human term placenta extract as detected by Western blot analysis (α-tubulin, internal control). (**D**) P-MSC (2 donors) expression of HLA-G protein isoforms after RNA silencing of HLA-G with small interfering RNA specific for HLA-G (siHLA-G) or non-target siRNA (siHigh GC), as measured by Western blot analysis (α-tubulin, internal control).

HLA-G has 7 isoforms, therefore we first delineated the pattern of *HLA-G* mRNA variants expressed by various sources of MSCs which were verified for minimal criteria of surface marker expression and trilineage differentiation criteria [29,30,31,32,33,34]. Nested RT-PCR analyses revealed that P-MSCs, hE-MSCs, and BMMSCs all express *HLA-G1, G3*, and *G4* mRNA variants, whereas the choriocarcinoma cell line JEG-3 can express all alternatively spliced *HLA-G* transcripts as expected (Figure 1A). In contrast, no expression of any *HLA-G* mRNA variants could be detected in the human choriocarcinoma cell line JAR, often used as a negative control in HLA-G studies [10], or human fibroblasts (cell line HS68). To ascertain for protein expression, we performed Western blotting using the MEM-G/1 antibody, which detects all HLA-G isoforms [35]. We found that BM- and P-MSCs express HLA-G1/G5 as well as a larger protein, approximately 70 kDa, which was also detected in JEG-3 cells (Figure 1B). In term placental tissue which serves as a positive control, all HLA-G protein isoforms could be detected except for HLA-G3 and a larger, approximately 50 kDa protein was also detected (Figure 1C). While these two higher molecular weight proteins have been previously been detected in human tumor exudates and identified as “HLA-G-like” molecules using a number of HLA-G antibodies for verification [36], we wish to ascertain this fact and thus performed knockdown with siRNA specific for HLA-G (Supplemental Figure S1). Specific knockdown of HLA-G expression in 2 donors of P-MSCs decreased expression of both the HLA-G1/G5 isoform as well as the larger 70 kDa isoform (Figure 1D). These findings demonstrate that diverse sources of human MSCs but not fibroblasts express 3 well-known *HLA-G* isoforms at the mRNA level, and 2 isoforms at the protein level: HLA-G1/G5 and a larger HLA-G-like molecule.

### 2.2. HLA-G Expression in BM & P-MSCs but Not JEG-3 Cells Is Responsive to IFN-γ Stimulation

**Figure 2:**
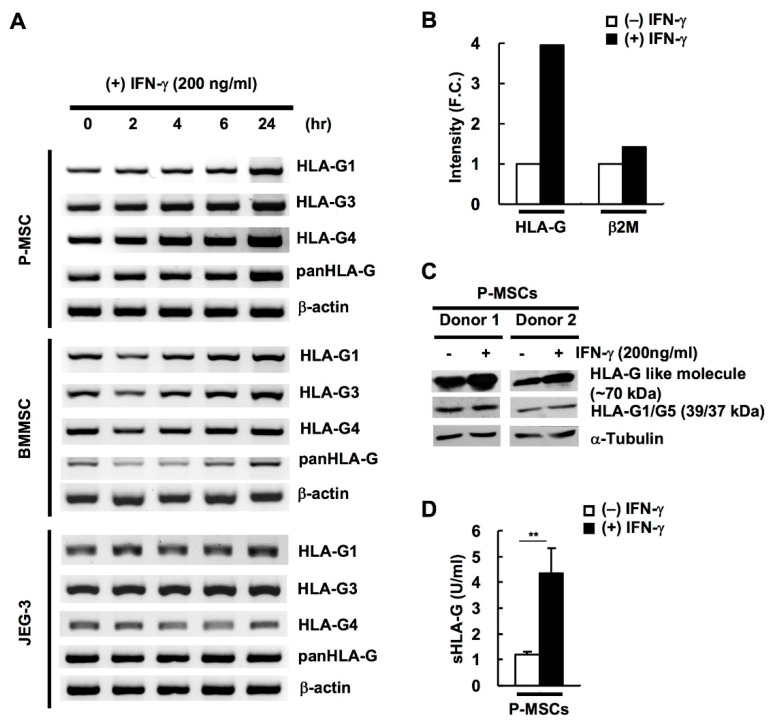
HLA-G expression in human MSCs but not JEG-3 cells is responsive to interferon-γ (IFN-γ) stimulation. (**A**) Expression of HLA-G transcripts (HLA-G1, G3, and G4) in IFN-γ-treated BMMSCs, P-MSCs, and JEG-3 cells at 0, 2, 4, 6, and 24 h as analyzed by nested PCR. β-actin was used as an internal control. Protein levels of various HLA-G isoforms in P-MSCs with or without IFN-γ treatment (200 ng/mL) as detected by (**B**) tandem mass spectrometric (MS/MS) analysis, (**C**) Western blot analysis, and (**D**) ELISA. ** *p* < 0.01.

IFN-γ is one of the most potent transcriptional inducers of MHC class I genes; however, HLA-G transcriptional responses to IFN-γ has been inconsistently reported, possibly due to the near exclusive use of cancer cell lines in these studies [37,38,39]. We found that IFN-γ treatment to P-MSCs and BM-MSCs led to increased expression of *HLA-G* at the transcriptional level as measured by nested RTPCR in a time-dependent fashion; in contrast, expression levels in JEG-3 remained largely constant over time (Figure 2A). To ascertain the increased gene expression resulted in modulation of protein expression, we performed MS/MS and immunoblotting in P-MSCs after IFN-γ treatment and found that HLA-G protein expression was increased, with the 70 kDa isoform more responsive than the 39/37 kDa (Figure 2B,C respectively). Moreover, ELISA demonstrated that sHLA-G expression in P-MSCs was also increased after IFN-γ stimulation (Figure 2D). Our data therefore demonstrate that IFN-γ can upregulate HLA-G mRNA and protein expression in human MSCs.

### 2.3. An Overall Hypomethylation Pattern Is Seen in the HLA-G Proximal Promoter CpG Sites of Human MSCs and Placental Tissue Extracts

**Figure 3:**
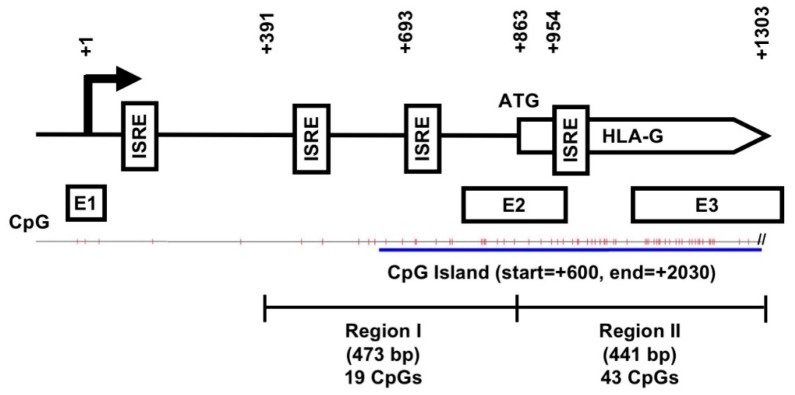
Schematic representation of predicted CpG site locations on the HLA-G proximal promoter and coding region. A large CpG island (blue line) extends from the promoter (+600) to Intron 4 of the gene body (+2030): Region I contains 19 CpG sites (tick marks) from +391 to ATG (+863) and Region II contains 43 CpG sites from ATG to +1303. Numbers indicated are relative to the transcription start site (TSS; +1). Boxes represent regulator binding sites: ISRE, interferon-stimulated response element; +1, transcription start site (TSS); ATG, translation initiation site; E, exon.

**Figure 4:**
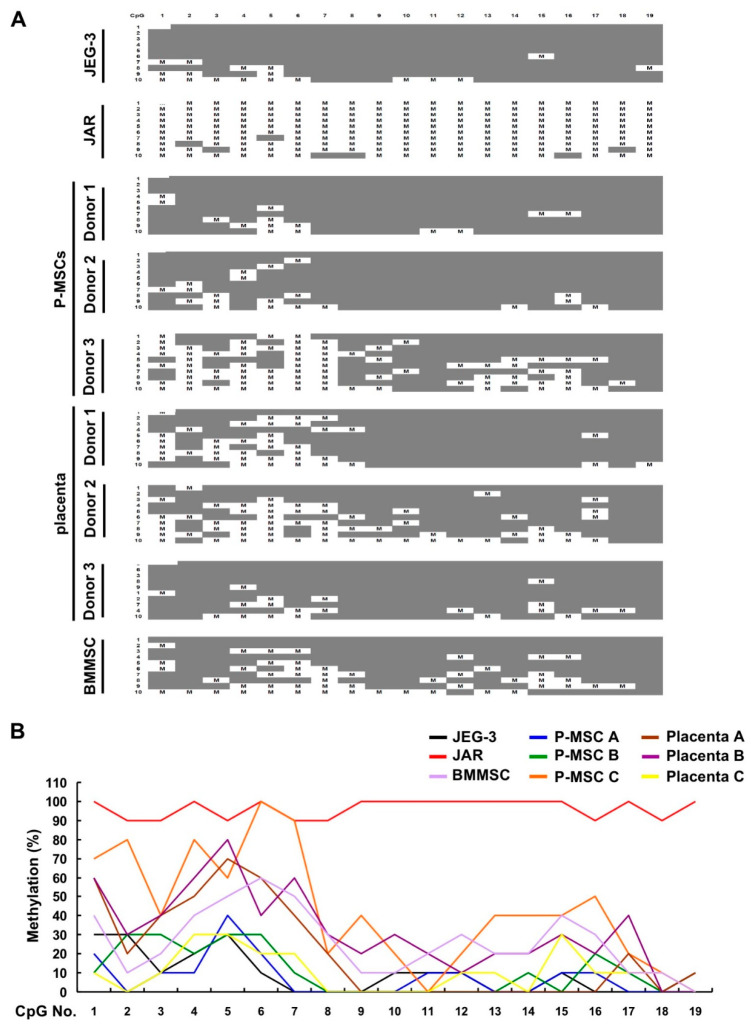
An overall hypomethylation pattern is seen in the HLA-G proximal promoter CpG sites of human MSCs and placental tissue extracts. (**A**) Region I (from +391 to ATG; 473 bp containing 19 CpG sites) of genomic DNA obtained from JEG-3, JAR, P-MSCs (3 donors), BMMSCs (1 donor), and placenta (3 donors) were sequenced after bisulfite modification with 10 bacterial clones of PCR products sequenced for each sample. Gray boxes, unmethylated CpG site; M, methylated CpG site. (**B**) Quantification of the percentage of individual CpG site which are methylated in Region I for each cell/tissue type. Methylation (%), percentage of total methylation per CpG site.

To assess for tissue-specific DNA methylation pattern in the proximal *HLA-G* promoter, we first used software to predict for CpG islands within this region and found a large CpG island extending from the promoter (+600) to Intron 4 of the gene body (+2030) (Figure 3). Interestingly, two ISRE sites were found within this CpG island, one in the proximal promoter region (at +693) which had been previously identified [24] and one within the gene body (at +954) now uncovered. Bisulfite sequencing was performed to determine the methylation profile of this CpG island in the proximal promoter region of the gene (Region I, covering 19 CpG sites from +391 to ATG). The overall pattern in this region was one of demethylation for P-MSCs and BMMSCs, as well as placenta tissue (Figure 4A). JEG-3 cells also demonstrated a strong pattern of demethylation, while in contrast, JAR cells demonstrated a pattern of near-complete methylation for all sites; these patterns for these 2 cell lines are in line with previous reports and consistent with their expression pattern for HLA-G, which is constitutive for JEG-3 cells and null for JAR cells [22,23]. Quantification of the range of site-specific methylation was approximately 0–10% in JEG-3 cells versus 90–100% in JAR cells, which correlated well with the HLA-G expression profile of these 2 cell lines (Figure 4B). P-MSCs, BMMSCs, and term placenta tissue, on the other hand, while demonstrating a highly diverse methylation pattern for the more distal CpG sites 1 to 7, only have approximately 30% methylation for the rest of the CpG sites, sites 8 to 19, which are also the sites most proximal to the start codon. Thus in 2 sources and multiple donors of human MSCs as well as term placenta, an overall pattern of hypomethylation is seen in the most proximal 12 CpG sites on the promoter.

### 2.4. CpG Sites within the Cell Body of HLA-G Are Strongly Hypomethylated in All Human MSCs and Placental Tissue but Nearly 100% Methylated in Both Choriocarcinoma Cell Lines

**Figure 5:**
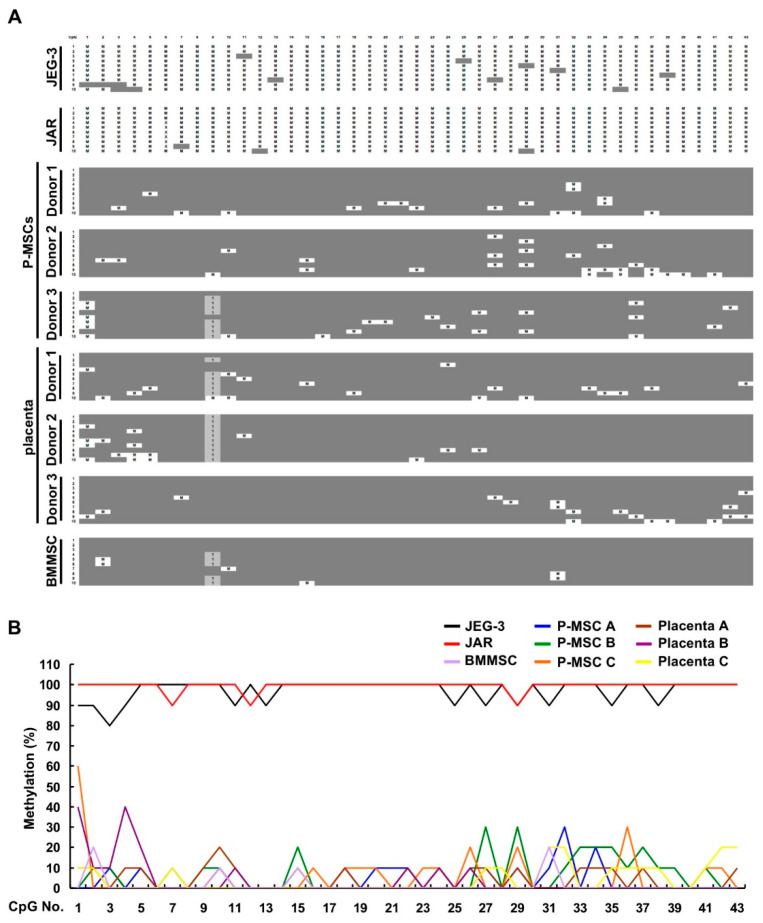
CpG sites within the cell body of HLA-G are strongly hypomethylated in all human MSCs and placental tissue but nearly 100% methylated in both choriocarcinoma cell lines. (**A**) Region II (from ATG to +1303; 441 bp containing 43 CpG sites) of genomic DNA obtained from JEG-3, JAR, P-MSCs (3 donors), BMMSCs (1 donor), and placenta (3 donors) were sequenced after bisulfite modification with 10 clones of PCR products sequenced for each sample. Gray boxes, unmethylated CpG site; M, methylated CpG site. X, missing CpG site (no.6); 1, CCGG → CCCG (CpG site no.9). (**B**) Quantification of the percentage of individual CpG site which are methylated within the HLA-G gene fragment spanning 441 bp downstream of ATG in each cell/tissue type. Methylation (%), percentage of total methylation per CpG site.

The extension of this CpG island from the proximal promoter into the gene body was intriguing to us, thus we continued evaluating the methylation pattern of the portion of the CpG island within the gene body of (genomic) *HLA-G*: from ATG to +1303 (Figure 5A). Interestingly, this portion of the CpG island contains 43 CpG sites which is nearly 3 times the amount of CpG sites found in the proximal promoter portion of the island. Surprisingly in this region, both JEG-3 and JAR cell lines exhibited a similar pattern of nearly 100% methylation at all CpG sites. On the other hand, very low levels of methylation were seen in all the normal cells/tissue, with 8 CpG sites (CpG No. 6, 8, 12, 13, 14, 17, 25, 30, and 40) being completely unmethylated. All normal cells and tissue but not the 2 choriocarcinoma cell lines demonstrated a nucleotide shift in CpG No. 9 (CCGG → CCCG) in 41% (29/70) of clones. In addition, a missing CpG site (No. 6) was seen only in JAR cells, in 60% (6/10) of the clones. Quantification of the range of site-specific methylation revealed an average of 0–20% in normal cells/tissues (Figure 5B). Overall, both primary human MSCs and placental tissue exhibit a strongly hypomethylated pattern in this gene body region of the CpG island, whereas the 2 choriocarcinoma cell lines are nearly 100% methylated.

### 2.5. Demethylation with 5-Azacytidine Results in Increased HLA-G Expression Patterns in MSCs

**Figure 6:**
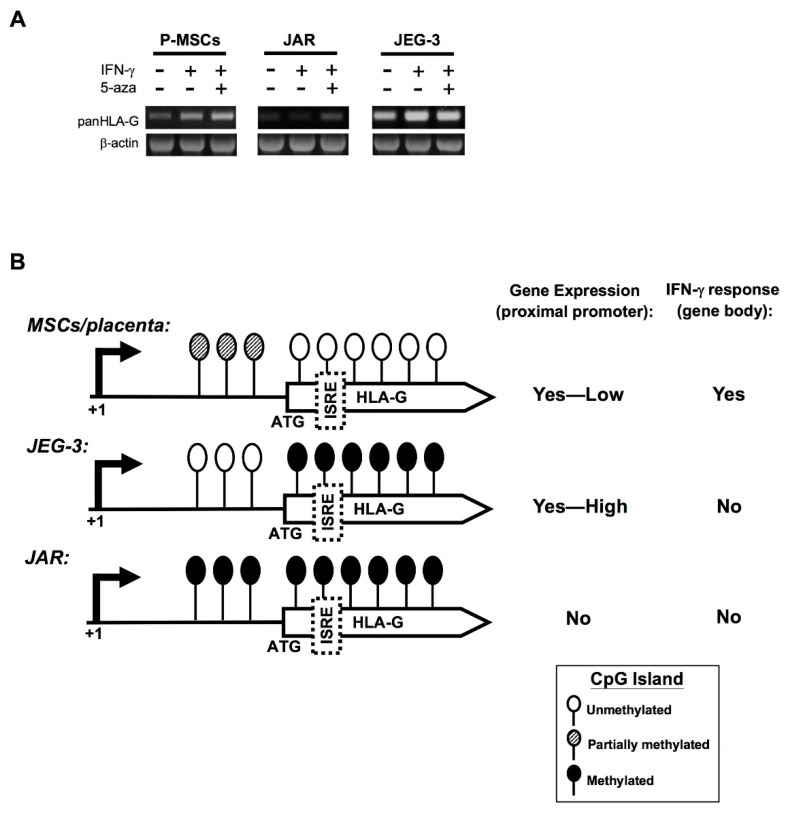
Demethylation with 5-azacytidine results in increased HLA-G expression patterns in MSCs. (**A**) RT-PCR analysis of HLA-G gene expression in P-MSCs (donor 1), JAR cells, and JEG-3 cells, after treatment with IFN-γ (200 ng/mL) and/or the demethylating agent 5-azacytidine (5-aza; 10 μm) after 24 h. (**B**) Proposed model of epigenetic regulation of HLA-G transcription in human MSCs and choriocarcinoma cell lines JEG-3 and JAR. Schematic representation of the unmethylated (white circles), partially methylated (striped circles), and methylated CpG sites (black circles) on the HLA-G proximal promoter and gene body in normal cells/tissue as represented by human MSCs and term placenta tissue, compared to the 2 choriocarcinoma cell lines JEG-3 and JAR, as correlated to HLA-G expression levels and IFN-γ responsiveness. ISRE (putative), interferon-stimulated response element; ATG, translation initiation site.

To assess how the different methylation patterns in the CpG island found in the primary human MSCs compared to the two cell lines affect gene expression, we treated all 3 cell types with 5-azacytidine (5-aza), a demethylating agent, and assessed for changes in *HLA-G* mRNA levels after 24 h. In P-MSCs at baseline, there is some level of *HLA-G* gene expression which is increased with IFN-γ treatment as expected even before 5-aza treatment, and these levels are further increased after 5-aza treatment (Figure 6A, left panel). In JAR cells which do not express HLA-G, IFN-γ treatment without 5-aza treatment did not result in gene expression, but after 5-aza treatment, low mRNA expression levels could be seen (Figure 6A, middle panel). In contrast, constitutive and high level expression of *HLA-G* was seen in JEG-3 cells regardless of 5-aza or IFN-γ treatments (Figure 6A, right panel).

The DNA methylation pattern in conjunction with the gene expression pattern obtained from the primary cells, tissue, and cancer cell lines lead us to formulate a model of HLA-G transcriptional control in different type of cells (Figure 6). Levels of DNA methylation within the promoter region correlate inversely with HLA-G gene expression levels: lack of methylation correlate with high expression levels as seen with JEG-3 cells, partial methylation correlate with low expression levels as seen in the normal, primary MSCs, whereas near-complete methylation correlate with no HLA-G expression as seen in JAR cells. On the other hand, the lack of DNA methylation within the gene body—in which a putative ISRE is located—which is only seen in normal cells/tissues but not the 2 choriocarcinoma cell lines, appears to correlate with a transcriptional response to IFN-γ. Thus, in summary, the unique promoter and gene-body DNA hypomethylation pattern of the HLA-G gene in human tissue-derived MSCs plays an important role in determining the baseline expression and IFN-γ responsiveness of HLA-G expression in these stem cells.

## 3. Discussion

HLA-G has long been known to impart immunomodulatory properties, but due to its restricted expression in few adult organs and transient tissues such as the placenta, understanding of its transcriptional regulation has proven difficult to achieve. The vast majority of studies have relied on using choriocarcinoma cell lines or overexpression systems, which do not represent normal cells or tissues and have raised skepticism at times [40,41,42]. Using two tissue sources of human MSCs as well as term placenta tissue, we profile the HLA-G isoforms which are expressed by these normal cells/tissues at the gene and protein levels, and found a large 70 kDa HLA-G-like isoform which was previously not identified to be expressed by MSCs but has been found in exudates from cancer patients [36]. Such HLA-G-like isoforms have also been identified to be ubiquitinated [43], and further studies are necessary to clarify whether these isoforms are physiologically relevant or cell stress markers. We also demonstrated that HLA-G expression is IFN-γ-responsive in 2 sources of human MSCs. Most critically, we found that epigenetic regulation in terms of unique DNA methylation pattern at the promoter region and within the gene body—including discovery of a putative ISRE (Figure 3)—are involved in HLA-G transcriptional control and responsiveness to IFN-γ in MSCs. Our findings, therefore, not only reveal new information on HLA-G transcriptional regulation, but also demonstrate the importance of using normal cells/tissue to elucidate molecular mechanisms involved in tissue-specific transcriptional control.

DNA methylation is known as an important epigenetic mechanism for transcriptional regulation, and found commonly at promoter regions to decrease transcriptional activity by impairing transcriptional initiation [28]. However, genome-wide analyses have now revealed that extensive DNA methylation exists in regions outside of proximal promoters including in gene bodies which have complex consequences on transcriptional activity [44,45,46]. We demonstrate that a CpG island which span the proximal HLA-G promoter also extend into the gene body, with a larger proportion within the gene body than the promoter region. Comparison of the methylation pattern within the promoter region and the gene body in normal cells, tissue, and 2 choriocarcinoma cell lines reveal distinct patterns which correlate with HLA-G transcriptional activity in the respective cell types. In the many studies using cell lines which reported a lack of functional IFN-γ regulatory elements in the HLA-G proximal promoter, one study presciently suggested that these elements may actually lie outside of the promoter region [37], which our data now support in primary human MSCs: a putative ISRE within the CpG island region of the gene body (Figure 6A). Our data on the methylation status of the HLA-G proximal promoter and gene body region in relation to its expression level in a specific cell type—the MSC—further contribute to understanding the complex role that DNA methylation has on tissue-specific transcriptional control. Our study does have limitations in that more donor samples and from other tissue sources of MSCs would allow for an even more comprehensive survey. In addition, more detailed molecular elucidation is required to establish clear mechanistic link between gene body methylation and tissue-specific HLA-G expression.

In summary, we profile the HLA-G isoforms expressed by various tissue sources of human MSCs and placental tissue, as well as validate the IFN-γ transcriptional responsiveness of HLA-G expression in these normal cells in contrast to choriocarcinoma cell lines, which have historically been used to study HLA-G transcriptional regulation. Compared to choriocarcinoma cell lines, MSCs and placental tissue revealed epigenetic regulation as evidenced by partial methylation at the proximal promoter region, and strong hypomethylation within the gene body region in which a putative ISRE was found, which may have contributed to the IFN-γ responsiveness of HLA-G transcriptional control in MSCs. Our study implicates the importance of using normal cells and tissues for physiologic understanding of tissue-specific transcriptional regulation, and the utility of human MSCs in understanding the transcriptional regulation of HLA-G to achieve better therapeutic application of this elusive but important immunomodulatory molecule.

## 4. Materials and Methods

### 4.1. Cell Culture

Human placenta-derived MSCs (P-MSCs) were isolated and expanded as described previously from term placentas (38–40 weeks gestation) obtained from healthy donor mothers after informed consent approved by the Institutional Review Board (CT9674) [20,47,48]. Human embryonic stem cells-derived MSCs (hE-MSCs) were derived as previously reported [21,31]. Characterized human BMMSCs were obtained from commercial sources (Promocell, Heidelberg, Germany) [29,31]. All MSCs were cultured in low-glucose DMEM (Gibco-Invitrogen, Carlsbad, CA, USA) with 10% fetal bovine serum (FBS; HyClone, Logan, UT, USA), 100 U/mL penicillin/100 g/mL streptomycin, and 2 mM L-glutamine (all from Gibco-Invitrogen) [47,49]. All MSCs were characterized for Minimal Criteria of trilineage differentiation and surface marker profile as established by the International Society for Cellular Therapy [31,32,33,34,50]. All procedures were done according to the Declaration of Helsinki guidelines. The human choriocarcinoma-derived cell lines JEG-3 and JAR, as well as the human fibroblast cell line HS68 were obtained from American Type Culture Collection (American Type Cell Culture, Manassas, VA, USA) and cultured according to manufacturer’s instructions. In some experiments, cells were treated with IFN-γ (200 ng/mL; R&D Systems, Minneapolis, MN, USA).

### 4.2. Reverse Transcription-Polymerase Chain Reaction (RT-PCR)

Total RNA was extracted using Trizol reagent (Gibco-Invitrogen), and RT-PCR was performed as reported previously with β-actin as internal control [48,51,52]. All primers including nested RT-PCR primers for various HLA-G mRNA isoforms are based on a previous report on human preimplantation embryos and inner cell masses [53].

### 4.3. RNA Silencing

RNA silencing was performed as reported previously [35], using HLA-G-specific small interfering RNA (siRNA; Stealth Select RNAi siRNA; Gibco-Invitrogen) along with manufacturer recommended negative-control siRNAs. siRNAs were transfected into cells by using Lipofectamine RNAiMAX (Gibco-Invitrogen).

### 4.4. Tandem Mass Spectrometric Analysis (MS/MS)

MS/MS analysis was performed as previously reported, using an LTQ-Fourier transformed cyclotron resonance mass spectrometer (Thermo Electron Corp., Waltham, MA, USA) with 1000 counts used as the minimum threshold for the cutoff for MS/MS sequential isolation [30].

### 4.5. ELISA

Secreted HLA-G (sHLA-G) production was measured by the sHLA-G-specific ELISA in accordance with the manufacturer’s instructions (Exbio Praha a.s., Vestec, Czech Republic) [54].

### 4.6. Western Blot Analysis

Protein was obtained from cells and Western blot analyses were performed as reported previously [52,55]. Primary antibody against all denatured HLA-G was detected with MEM-G/1 (Exbio) [35], with primary antibody against α-tubulin (Santa Cruz Biotechnology, Santa Cruz, CA, USA) was used as internal control [52].

### 4.7. Genomic DNA Extraction and Bisulfite Modification

CpG sites and island were identified with the UCSC Genome Browser Gateway (http://genome.ucsc.edu/cgi-bin/hgGateway). Cultured cells and placental tissue were lysed with TE buffer supplemented with 1% SDS, 0.4 mg/mL RNAse A (Gibco-Invitrogen) and 0.2 mg/mL proteinase K (Sigma-Aldrich, St. Louis, MO, USA). Genomic DNA was phenol-extracted and ethanol precipitated. Bisulfite modification was performed using the EZ DNA Methylation-Lightning kit (Zymo Research, Irvine, CA, USA) according to the manufacturer’s instructions. Bisulfite converted DNA was amplified by EPIK Amplification kit (Bioline, London, UK) with bisulfite conversion specific primers (modified from [23]) listed in Table 1. PCR fragments were cloned into the pGEM-T Vector System (Promega, Madison, WI, USA) and subjected to sequencing analysis (Genomics, New Taipei City, Taiwan).

### 4.8. Statistical Analysis

Statistical analysis was performed with the Student’s *t* test for analysis between two groups, and ANOVA for analyses of multiple groups using GraphPad Prism software (GraphPad Software Inc., La Jolla, CA, USA). All data are presented as mean ± SEM, with *p <* 0.05 considered statistically significant.

## Figures and Tables

**Table 1:** List of Primers.

Primer	Sequence	Product Size
Region 1-Forward	AAGAGTATAGGAGGATAGGTAAGG	449 bp
Region 1-Reverse	TGGGGAGAATGAGTTTGGGTGGG	
Region 2-Forward	GTTAAGGATGGTGGTTATGG	447 bp
Region 2-Reverse	AAATTCATTCTATCAATCTATAC	

## Supplementary Materials

The following are available online at https://www.mdpi.com/1422-0067/21/14/5075/s1, Figure S1. Gene and protein expression of HLA-G isoforms in JEG-3 cells after RNA silencing of HLA-G with small interfering RNA specific for HLA-G (siHLA-G) or non-target siRNA (siHigh GC) as analyzed by RT-PCR and western blot.
