# Supplementary material for: HLA-G Expression in Human Mesenchymal Stem Cells (MSCs) Is Related to Unique Methylation Pattern in the Proximal Promoter as well as Gene Body DNA
Source: Int J Mol Sci. 2020 Jul 18;21(14):5075. doi: 10.3390/ijms21145075 (PMC7404323; doi:10.3390/ijms21145075)
Supplement: Supplementary file 1 [file ijms-21-05075-s001.zip › ijms-848223-supplementary.docx]

Supplementary material


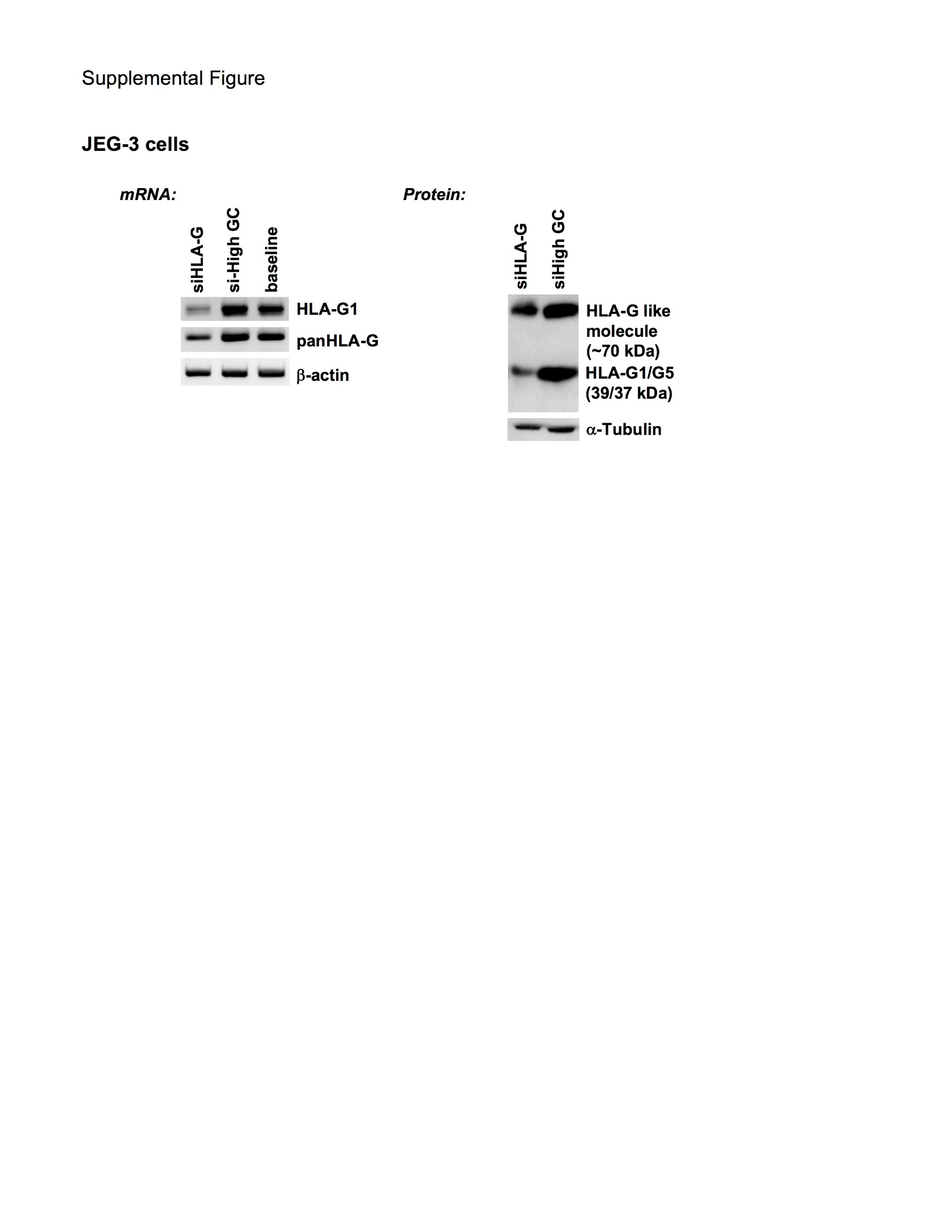


**Figure S1.** Gene and protein expression of HLA-G isoforms in JEG-3 cells after RNA silencing of HLA-G with small interfering RNA specific for HLA-G (siHLA-G) or non-target siRNA (siHigh GC) as analyzed by RT-PCR and western blot, respectively.
